# Open Science in Undergraduate Medical Education: An International Framework for Transparency, Responsibility, and Professional Identity Formation

**DOI:** 10.5334/pme.2600

**Published:** 2026-09-30

**Authors:** Haang Jeung-Maarse, Rosa L. Ecos, Luisa F. Jimenez-Soto, Philipp Linde, Mara Müssigmann, Sandra Schuh, Sarah Wahlster, Changryul Claud Yi

**Affiliations:** 1 Department of Psychiatry and Psychotherapy, Protestant Hospital Bethel Foundation, Medical Faculty OWL, Bielefeld University, Bielefeld, Germany; 2 National Institute of Neurological Sciences, Lima, Peru; 3 Walther-Straub Institute of Pharmacology and Toxicology, Medical Faculty, LMU Munich, Munich, Germany; 4 Department of Radiation Oncology, Cyberknife and Radiation Therapy, Faculty of Medicine and University Hospital of Cologne, University of Cologne, Cologne, Germany; 5 Institute of Medical Education, LMU University Hospital, LMU Medizin, Ludwig-Maximilians-Universität München (LMU Munich), and Module Coordinator for the Scientific Curriculum (MeCuM Science, Module 6) and the Practical Year (Praktisches Jahr, PJ), Faculty of Medicine, Munich, Germany; 6 Department of Dermatology and Allergology, University Hospital Augsburg, Augsburg, Germany; 7 Faculty of Medicine, Medical Didactics and Educational Research (DEMEDA), Augsburg, Germany; 8 University of Washington, Seattle, USA; 9 Department of Plastic and Reconstructive Surgery, Pusan National University School of Medicine and the Biomedical Research Institute, Pusan National University Hospital, Busan, Republic of Korea

## Abstract

Concerns about reproducibility, transparency, and research waste have intensified debates about trust in medical research and health-related sciences. Although Open science initiatives aim to address these challenges, their role in undergraduate medical education remains underexplored. Undergraduate medical education typically emphasizes appraisal of published evidence but pays less attention to how research is conducted, governed, and disseminated. This article proposes an international conceptual framework that positions Open science as part of professional formation in undergraduate medical education. The framework comprises three domains: epistemic transparency, ethical responsibility, and professional identity formation. Together, they describe how learners engage with knowledge production, uncertainty, and responsibility. Rather than prescribing curricular models, the framework offers adaptable principles that can be mapped onto existing structures such as competency-based programs in diverse contexts. By framing Open science as a formative orientation rather than a technical skill set, it links medical education to professional responsibility and the public role of medicine.

## Introduction

### Reproducibility Crisis, Trust, and Medical Education

Concerns about reproducibility, transparency, and research waste have raised fundamental questions about how medical knowledge is generated, evaluated, and translated into clinical practice [1]. In a 2024 survey of more than 1,600 biomedical researchers, 72% reported a reproducibility crisis. Respondents identified pressure to publish and the prioritization of novelty over replication as key contributors [2]. Only 16% reported institutional procedures supporting reproducibility, suggesting that irreproducibility is widely perceived as a systemic and cultural challenge rather than a purely technical limitation. At the same time, global health crises have intensified public scrutiny of scientific processes and amplified debates about trust and the societal legitimacy of medical research, particularly during the COVID-19 pandemic [3].

These developments have important implications for undergraduate medical education. Physicians are expected to critically appraise evidence, practice evidence-based medicine, and contribute responsibly to knowledge production. At the same time, they must maintain public trust in an era of rapid scientific change and heightened societal expectations, including in global health challenges [4]. These competencies depend on understanding how medical knowledge is generated and should therefore be developed early in undergraduate medical education, with further consolidation during dedicated research training. Yet undergraduate medical education rarely addresses the structural conditions under which research findings can be considered reliable. While evidence-based medicine training emphasizes interpreting published results, it often neglects how those results are generated, documented, and governed. In this sense, research training in medical school is not only about technical skills, but about preparing future physicians to participate responsibly in the production and stewardship of medical knowledge.

At the same time, undergraduate curricula face increasing pressures of competing content and increasing complexity. Within competency-based medical education, the CanMEDS framework has become a canonical way of summarizing what physicians are expected to know, do, and be, by articulating seven interdependent roles (Medical Expert, Communicator, Collaborator, Leader, Health Advocate, Scholar, and Professional) that together define competent practice [5]. This role-based summary provides an important high-level structure, yet the amount of factual knowledge and technical skill that could be linked to each role continues to grow, contributing to perceptions of curriculum overload and to debates about what should count as core content in undergraduate programs. Within this landscape, there is a need not only for additional content but for overarching concepts and values that can help organize existing curricular elements across roles.

Open science can serve such a function by connecting questions of how knowledge is produced and shared (Scholar), how physicians act within systems and toward communities (Leader and Health Advocate), and how they understand their ethical and professional commitments (Professional), thereby offering a unifying lens rather than another discrete content area. Elements of these domains are already present in most undergraduate curricula (e.g., research methods and evidence-based medicine [6], ethics and professionalism teaching [7], and longitudinal role modelling [5]), but they are rarely connected explicitly to how medical knowledge is produced and governed or framed in relation to Open science principles.

More broadly, the Open science movement responds to these challenges by promoting accessibility, transparency, and reproducibility in research [8]. Its practices include preregistration, Open access dissemination, transparent reporting standards, and structured data stewardship. The FAIR principles define data as findable, accessible, interoperable, and reusable [9]. These practices aim to strengthen the verifiability and integrity of scientific knowledge. Open science is increasingly discussed alongside Open education, which promotes openly accessible, adaptable resources and pedagogies that foreground equity and social justice [10]. In undergraduate medical education, these ideas matter for all future clinicians who must interpret evidence, communicate uncertainty, and act responsibly toward patients, colleagues, and the public [11].

However, the Open science ecosystem reflects global inequalities [12]. Differences in governance structure, academic infrastructure, as well as research regulation, mentorship, funding, and protected research time between high- and low-income countries contribute to unequal implementation [13]. As recent work on Open science and Open education has shown, open practices are not equally accessible or risk-free for all; therefore, minoritized and underserved populations and those in resource-constrained settings may face barriers when engaging in openness [14]. Without contextual sensitivity, Open science education risks reinforcing existing disparities in knowledge production [15].

### Educational Gap

Open science principles remain only sporadically integrated into undergraduate medical education [16]. Existing initiatives are often local and exploratory. A student-led initiative in Uganda, for example, promoted Open access, Open educational resources, and open data through multi-stakeholder engagement [17], illustrating emerging interest and advocacy rather than formal curricular implementation. While such efforts demonstrate growing grassroots interest in Open science, they also highlight structural and infrastructural constraints that limit sustained institutionalization in resource-limited settings. Systematic, transferable frameworks for integrating Open science into undergraduate medical curricula across diverse contexts remain largely absent.

This gap is also reflected in the variable place of scholarly training within undergraduate medical curricula. Across institutions and countries, undergraduate scholarly training ranges from structured research tracks to minimal exposure confined to statistics or evidence-based medicine courses.

In Germany, for example, an analysis of doctoral regulations across all medical faculties revealed substantial heterogeneity in requirements for the medical thesis (“Doktorarbeit”), including variation in publication expectations, supervision agreements, formal training in Good Scientific Practice, and plagiarism screening [18]. In Germany, the “Doktorarbeit” is usually undertaken during or shortly after undergraduate studies, whereas comparable research experiences may occur at other stages elsewhere. This example illustrates a broader point: competencies in reproducibility, data stewardship, and transparent research practices are often acquired unevenly and informally rather than through clearly structured educational pathways.

Evidence from psychology and the life sciences suggests that barriers to researchers’ adoption of Open science practices, such as open publishing and data sharing, are primarily educational and institutional [19,20,21]. These findings concern research practice rather than the application of Open science in medical education. Reported barriers include insufficient training, uncertainty about legal and ethical frameworks, limited institutional support, and misaligned incentives. For undergraduate medical education, this implies that Open science should be introduced early and explicitly, rather than being left to optional research experiences or informal socialization, in line with IFMSA’s call to embed Open science and Open education principles into medical curricula rather than treating them as extracurricular activities [11]. If these gaps persist, training in research integrity will remain uneven and may ultimately weaken the credibility of future medical practice. This highlights an urgent need for an international framework that identifies shared principles while permitting context-sensitive implementation. Although educational structures differ widely, undergraduate medical students must already learn to find reliable information and distinguish robust from poor-quality research, and many will later, as physicians, contribute to the production and stewardship of knowledge. Open science, by emphasizing transparent generation and communication of knowledge, directly links professional identity formation to public trust in medicine.

Building on this cross-cutting lens, the framework proposed in this paper does not replace existing competency frameworks such as CanMEDS. Instead, it uses Open science to highlight three interrelated domains that run across these roles: transparency, responsibility, and professional identity. These domains were chosen because Open science practices make knowledge-generating processes more visible (transparency), emphasize the ethical and societal obligations of researchers and institutions (responsibility), and influence how future physicians develop their professional values, roles, and commitments (professional identity).

The central contribution of this paper is to conceptualize Open science as an essential dimension of professional formation in medicine rather than merely a set of research practices. We propose that integrating Open science into routine educational and clinical activities, and into assessment and learning culture, is essential to normalize transparency and accountability as core professional norms. We introduce a framework structured around three domains, namely epistemic transparency, ethical responsibility, and professional identity formation, to guide curricular development, educational research, and institutional reflection in undergraduate medical education. Rather than prescribing specific curricula, it offers shared principles that support context-sensitive integration of Open science across diverse educational and institutional settings.

## Conceptual Framework

Open science is often treated as a set of tools and infrastructures, such as repositories, data-sharing platforms, and open-access publishing workflows, which risks leaving it peripheral to professional formation. In our framework, it instead functions as a formative orientation that shapes how future physicians interpret evidence, handle uncertainty, and take responsibility for medical knowledge. Engagement with how knowledge is produced is a core professional responsibility for all physicians, not only those pursuing research careers. It therefore belongs within undergraduate medical education, where foundational professional habits and orientations are formed. Addressing these challenges calls for integrating Open science into professional values and scholarly habits, rather than limiting it to technical instruction.

We propose a flexible international framework structured around three domains: epistemic transparency, ethical responsibility, and professional identity formation (Figure 1). Together, these domains embed Open science within professional formation and link transparency to accountability in medical practice.

**Figure 1:**
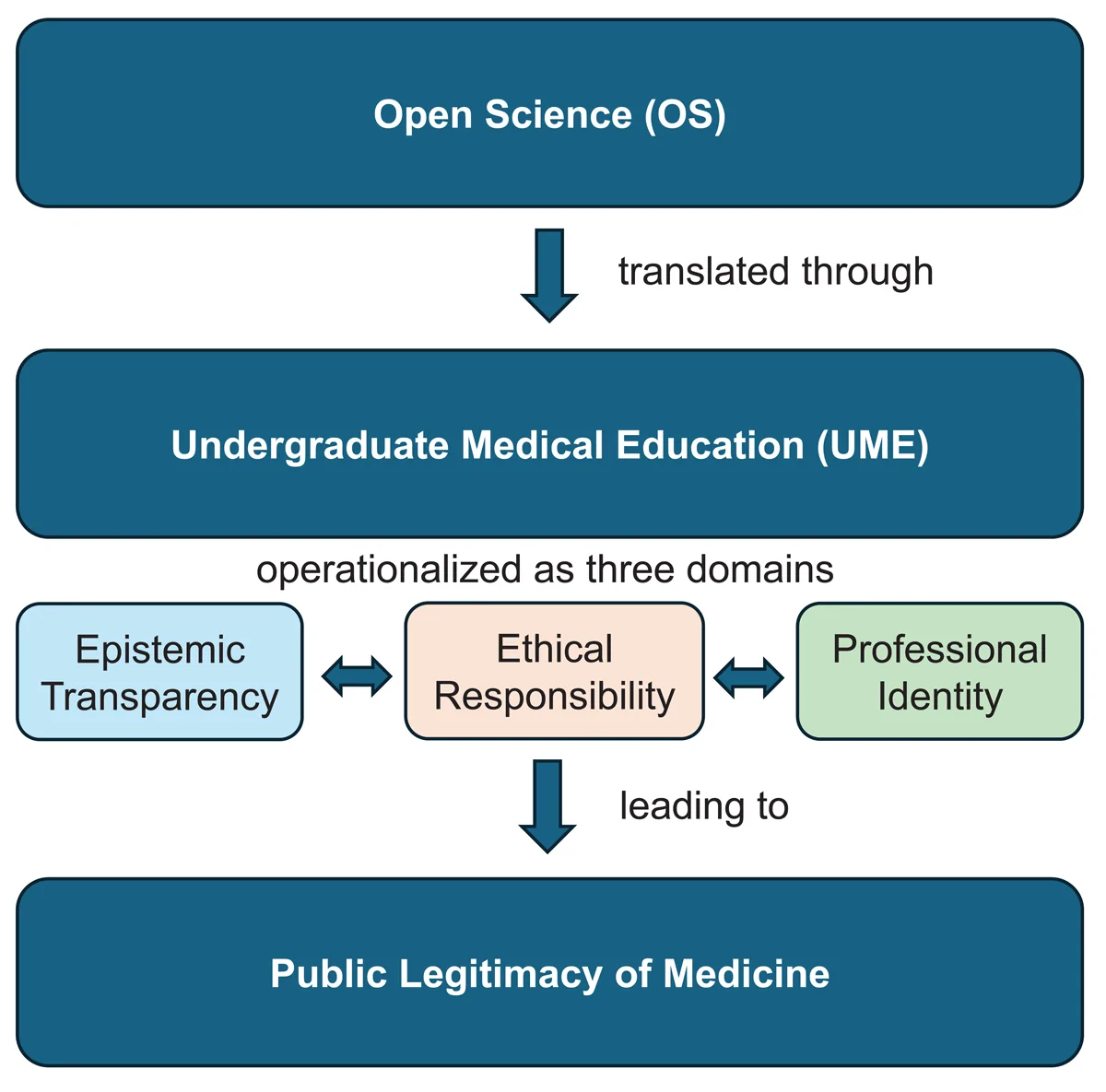
Conceptual framework linking epistemic transparency, ethical responsibility, and professional identity formation in Open science.

### Epistemic Transparency

Epistemic transparency refers to making visible how medical knowledge is generated, justified, and communicated. In undergraduate medical education, this means going beyond teaching what is known to also explain how knowledge is produced, including the assumptions, methods, uncertainties, and limitations that shape it. Learners engage not only with research findings but also with the processes and reasoning behind them. Making these processes explicit supports a more critical and reflective understanding of medical knowledge. For example, learners may examine how study design, data interpretation, and publication practices influence what is accepted as evidence, and how these features shape the way evidence is communicated to patients, communities, and other publics.

Persistent concerns about irreproducibility, selective reporting, and research waste show that credibility depends not only on findings but also on whether methods and analytic decisions are transparent and can be reconstructed [22]. In medical education, fostering epistemic transparency therefore demands more than teaching critical appraisal of published results. It involves cultivating both an Open science mindset and a corresponding skill set. The mindset entails reflective awareness of how academic incentives, publication norms, and performance metrics shape knowledge production and public trust. The skill set translates this awareness into practice through engagement with core domains such as open access, data and materials sharing, reproducible analyses, preregistration, and replication research [23].

Educationally, epistemic transparency involves reorienting scholarly habits. This includes making assumptions explicit, documenting analytic reasoning, and recognizing transparency signals in published work [23]. In competency-based medical education, where large volumes of clinical and educational data are generated, transparent data governance and responsible data sharing become institutional as well as individual responsibilities [24]. Digital Open science initiatives illustrate how reproducible workflows and shared infrastructures can support such practices, and Toelch & Ostwald [25] illustrate examples of tools and teaching formats that enable transparent and reproducible research workflows in practice. However, tools alone are insufficient without pedagogically designed support [21]. Linking these principles to data literacy prepares students for increasingly data-rich clinical environments [26]. Open educational practices, such as involving students in adapting or creating openly licensed learning materials or public-facing resources, can further connect epistemic transparency to how knowledge is shared with peers, patients, and the wider public [27,28,29]. Examples include the Free Open Access Medical Education (FOAMed) movement, which provides openly accessible, collaboratively produced educational resources that complement traditional curricula and model participatory approaches to medical knowledge [30].

### Ethical Responsibility

Ethical responsibility addresses the moral dimensions of knowledge production and dissemination and represents a second foundational domain of Open science in medical education. A central objective is to frame openness as a spectrum of accountable disclosure rather than a binary state. Decisions about consent, data stewardship, authorship, access, and dissemination are not merely procedural. They reflect commitments to fairness, accountability, and respect for participants. Open science does not replace research ethics; rather, it makes ethical decision making more explicit and therefore more teachable.

Extending established clinical principles such as confidentiality, beneficence, and justice to research practice reinforces continuity between clinical and scholarly responsibility. Within this framework, transparency is understood as an ethical obligation that must be balanced with autonomy, privacy, and institutional regulation. The guiding norm of “as open as possible, as closed as necessary” frames openness as a justified ethical choice [15].

Educationally, undergraduate medical students engage with scenarios such as dual-role tensions, secondary data use, and multi-site collaboration [31]. Using a simplified ethics application format, learners map the research lifecycle from protocol development through consent, data handling, and dissemination. They identify concrete safeguards, including minimal risk justification, consent language addressing potential future reuse, de-identification strategies, secure storage, and governance for access decisions [32]. Open science tools such as role-based project permissions and tiered data availability statements are introduced as accountability mechanisms that support responsible transparency [33]. By linking ethical reflection with practical governance strategies, this domain prepares students to align openness with participant protection and institutional requirements. In doing so, ethical responsibility operationalizes Open science as professional integrity enacted within real-world constraints.

### Professional Identity Formation

Medical education has increasingly been described as a process of professional identity formation rather than solely the acquisition of competencies [34]. This process begins as students first encounter clinical environments and start to articulate what kind of physicians they aim to become. Professional identity develops as learners internalize values, norms, and responsibilities through socialization, role modeling, and participation in authentic professional practice [35]. When transparency, reflexivity, and accountability are presented not only as ethical responsibilities but as core professional virtues, they become embedded in how future physicians understand their role.

Establishing these values extends beyond formal instruction, particularly in early training [36]. It depends on their consistent enactment across learning environments. Role modeling by clinicians and educators is central, as learners observe how uncertainty is communicated, how evidence is interpreted, and how decisions about data sharing and reporting are justified in practice. Structured opportunities for reflection further support this process by encouraging learners to examine tensions between ideals of openness and the practical constraints of clinical work.

Embedding Open science within routine educational and clinical activities, such as case discussions, research projects, and quality improvement initiatives, helps normalize transparency as part of everyday professional practice. Assessment and feedback can reinforce this by valuing not only correct outcomes but also the clarity and accountability of reasoning. When institutions and clinical environments consistently model and reward transparency, accountability, and responsible knowledge practices, these values become part of the professional norms that learners internalize.

Open science contributes to this formation by positioning physicians not only as consumers of evidence but also as stewards and communicators of knowledge. Clinicians interpret and apply guidelines, conduct consultations, teach students and colleagues, explain uncertainty to patients, and participate in public discourse [37]. In each of these roles, the ability to articulate how knowledge was generated, what its limitations are, and how uncertainty should be understood becomes central, directly linking transparency to professional identity and public trust. By embedding transparency within professional formation, medical education strengthens medicine’s legitimacy as a socially accountable and self-regulating profession.

Despite these educational aims, structural tensions may limit their realization. Promotion and reward systems in academia often prioritize publication output, creating incentives that may conflict with principles of transparency, accountability, and responsible knowledge production [2]. As a result, learners may encounter discrepancies between formally taught values and practices observed in clinical and research environments. Within the framework, these tensions signal that curricular efforts need to be accompanied by institutional reflection on recognition and reward structures, so that the values associated with Open science, responsibility, and professional identity are not only taught but also visibly enacted and rewarded.

## Discussion

### Reframing the Problem: From Research Reform to Educational Formation

Integrating Open science into undergraduate medical education reframes how future physicians engage with medical knowledge. Although debates about reproducibility and transparency are often framed as methodological or policy challenges, they are increasingly recognized as systemic and cultural issues within scientific practice [8,38] that must also be addressed through undergraduate medical training. Rather than adding a separate Open science module to already dense programs, educators can use the framework to ensure that epistemic transparency, ethical responsibility, and professional identity formation are taught as core principles that permeate existing teaching, assessment, and scholarly activities, while using it to identify gaps and redundancies and to introduce targeted enhancements within current curricular structures.

Positioning Open science within undergraduate education shifts attention from later compliance to early professional formation. Sustainable change depends not only on improved infrastructures or incentives but also on how learners are socialized into norms of accountability, transparency, and responsible knowledge stewardship. Embedding these orientations early in training helps integrate them into future clinical and academic practice rather than leaving them as external requirements. In this sense, Open science becomes part of “who physicians are” and “how they think” about knowledge, rather than an add-on technique to be applied selectively.

### Professional Identity Formation and the Epistemic Dimension of Practice

This reframing aligns with perspectives that understand medical education as professional identity formation rather than solely competency acquisition [34]. In this view, learning how knowledge is generated and justified becomes part of becoming a physician rather than an optional scholarly specialization.

Contemporary clinical practice asks physicians to interpret rapidly evolving evidence, communicate uncertainty, and justify decisions in contexts shaped by public scrutiny and information abundance [39]. The growing use of data-driven tools and artificial intelligence further amplifies these demands and makes it more difficult to assess the reliability and provenance of knowledge. In this context, epistemic transparency becomes a professional capability rather than a purely methodological concern.

Framing engagement with uncertainty and the provisional nature of knowledge as normal aspects of medical practice may help position transparency not as a weakness but as an expected feature of professional reasoning. In this sense, Open science extends professional responsibility into the epistemic domain, linking how knowledge is produced to how it is used in clinical care. This linkage underpins the framework’s emphasis on transparency, responsibility, and professional identity formation as mutually reinforcing domains rather than separate teaching topics.

### Educational Implications for Curricular Integration

Viewing Open science as a dimension of professional formation has implications for how it is taught within already packed curricula. Rather than introducing isolated courses or technical workshops, integration should occur longitudinally across existing learning environments and be treated as a core principle that permeates teaching, assessment, and learning culture.

A practical first step is to use the framework as a mapping tool. Programs can review where existing learning activities already address how knowledge is produced, how ethical decisions around data and dissemination are made, and how professional values related to openness and accountability are modeled, assessed, and reinforced. Curriculum mapping, already used in health professions education to identify gaps, overlaps, and alignment between competencies, learning opportunities, and assessment [40], offers a feasible strategy for integrating Open science without simply adding more content.

Concrete entry points can then be aligned with each domain. Within epistemic transparency, brief protocol-writing exercises [41] and exposure to preregistration formats [42] help students understand how trustworthy evidence is generated and how transparent workflows support critical appraisal and accountability. Within ethical responsibility, Open science can be embedded into existing ethics and professionalism teaching through cases on consent for secondary data use, data sharing and privacy, authorship, access inequities [43,44], and the use of Open educational resources and Open pedagogy (e.g. students co-creating or adapting openly licensed materials) [30]. Within professional identity formation, reflective writing [36], mentoring conversations, portfolios, and case discussions can make explicit how openness, reflexivity, error correction, and communication of uncertainty relate to being a trustworthy physician [45,46]. These efforts are unlikely to succeed without supportive faculty development and institutional alignment, because learners infer norms not only from formal teaching but also from what teachers model and what institutions reward [28,47].

Educational research has long emphasized the influence of the hidden curriculum and the importance of role modeling in shaping professional values [48,49]. These tensions around recognition and reward systems further illustrate that efforts to support Open science-related professionalism require institutional as well as curricular change. Integrating Open science therefore requires engagement not only with students but also with educators and clinical supervisors, ensuring that transparency and responsible knowledge practices are reinforced across contexts. A principle-based approach allows adaptation to diverse educational systems while maintaining shared commitments to accountability and responsible knowledge production.

### Global and Contextual Considerations

Open science is not equally accessible or achievable for all researchers. Without attention to contextual realities, Open science initiatives risk reinforcing existing inequities in knowledge production [12]. Differential barriers related to career stage, institutional resources, geographical location, and research infrastructure mean that underserved and marginalized groups may face disproportionate risks and fewer benefits when engaging in open practices. Naming these constraints is important, but so is focusing on ways to mitigate them, for example, by leveraging technology such as tele-medicine, virtual education, and digital collaboration platforms to build partnerships that share expertise and resources across settings.

In a world marked by inequities in health outcomes, workforce distribution, and access to high-quality education, medical education has a responsibility to foster critical appraisal of evidence, attention to structural determinants of health, and a commitment to social accountability across local and global settings. Aligning undergraduate medical education with Open science principles can support these aims by promoting equitable access to knowledge, transparent reasoning about uncertainty, and more inclusive and bidirectional forms of knowledge production, while remaining attentive to digital and infrastructural divides that risk exacerbating existing inequalities. The international development of this framework reflects both shared aspirations and contextual variability in Open science education: collaborators across regions identified common principles but also differences in infrastructure, mentorship, regulatory environments, and digital access, underscoring the need for flexibility and caution against assuming uniform implementation pathways.

Although Open science has become increasingly institutionalized within universities worldwide, these developments have largely focused on research practices rather than the formation of future professionals. Positioning this framework within that landscape suggests that medical education may represent a critical next step in the maturation of the Open science movement. In Europe, three dominant models can be observed: In a strategic identity model, Open science is foregrounded as a core institutional value and branding element, aligned with broader visions of responsible research and education (e.g., Utrecht University) [50]. In an infrastructure model, universities invest primarily in platforms, services, and specialist centres that support reproducible and transparent research (e.g., LMU Munich Open Science Center [51] and the Open Science Initiative in Medicine [52]. In an alliance model, Open science is advanced through interinstitutional consortia that coordinate policies, infrastructures, and incentives across universities (e.g., Berlin University Alliance) [53]. Outside Europe, similar developments exist but are often driven by different mechanisms. In the United States, universities have expanded reproducibility and open research programs largely in response to federal mandates around data management, public access, and research transparency [54]. In Latin America, infrastructures such as the Scientific Electronic Library Online (SciELO) and the Red de Revistas Científicas de América Latina y el Caribe, España y Portugal (RedALyC) reflect long-standing regional commitments to publicly funded, non-commercial Open access publishing and community-governed platforms [55]. In Africa, efforts to institutionalize Open science emphasize the creation of enabling policies, infrastructures, and incentive structures in resource-constrained settings, with particular attention to funding, leadership, and capacity building at institutional and national levels [56]. Across these diverse models, formal attention to Open science within undergraduate and postgraduate medical curricula remains limited, underscoring the need for context-sensitive educational approaches that can complement institutional and policy-level initiatives.

### Implications for Trust and the Societal Role of Medicine

Surveys suggest that physicians remain among the most trusted professionals worldwide [57], yet confidence in doctors and hospitals has declined in several countries since the COVID-19 pandemic, with lower trust concentrated in socioeconomically disadvantaged and politically marginalized groups [3]. Physicians play a central role in interpreting evidence, communicating uncertainty, and explaining the limits of knowledge to patients and the public, and thereby in enacting medicine’s broader societal role as an accountable mediator between scientific knowledge, health systems, and communities. A critical scoping review of patient–physician verbal communication identifies clear and explicit language, patient activation, negotiation of epistemic authority, affiliative language, and attention to both transactional and relational goals as key conceptual dimensions for effective encounters [58]. An Open-science-aligned curriculum can operationalize these insights by giving learners practice in explaining how evidence is generated in plain language, inviting patients into discussions about options, and explicitly negotiating “who knows what” and how certain that knowledge is. Finally, studies indicate that visible open-science practices, such as sharing data and materials, preregistering studies, and providing open access to research, can improve laypeople’s perceptions of scientific trustworthiness, particularly when the purpose of these practices is communicated [59]. Taken together, these developments position Open-science-aligned medical education as one concrete way for the profession to fulfil its societal responsibility to maintain trust and promote more equitable access to trustworthy knowledge across diverse patient groups.

### Limitations and Future Directions

This framework represents a conceptual synthesis rather than an empirical evaluation, and its educational impact remains to be tested. Implementation will vary across institutions, and the proposed domains are intended to guide reflection rather than prescribe specific curricular formats or assessment strategies.

Practical constraints must also be considered. Integrating these concepts into already dense curricula requires careful prioritization, and early learners may have limited prior knowledge to engage with complex epistemic and ethical questions. Emerging technologies, including artificial intelligence, may further complicate the implementation of Open science principles by introducing new challenges related to transparency, verification, and interpretation.

Future research should examine how these principles are operationalized in different educational contexts, how educators and learners experience their integration, and whether they influence professional attitudes, research behavior, and engagement with scientific knowledge over time, while acknowledging that robust assessment of long-term real-world impact will be challenging and complex.

## Conclusion

This article proposes an international framework that positions Open science as a component of professional formation in undergraduate medical education. By linking epistemic transparency, ethical responsibility, and professional identity formation, it reframes undergraduate medical training in research as preparation for responsible participation in knowledge production rather than the acquisition of technical skills alone.

The framework offers adaptable, principle-based guidance for educators designing and refining medical curricula for diverse educational contexts while maintaining shared commitments to accountability and social responsiveness. Failing to integrate Open science into medical education risks perpetuating opaque research practices and undermining trust in medical knowledge. Integrating Open science into medical education is therefore both a response to concerns about reproducibility and an opportunity to strengthen transparency and responsibility as a defining feature of contemporary medical professionalism. Future research should examine how these principles are implemented across different curricular structures, and how they shape learners’ professional development and engagement with scientific knowledge, and how institutional cultures and recognition systems enable or constrain their enactment.

## Artificial Intelligence

Artificial intelligence tools were used to support language editing and stylistic refinement of the manuscript. All conceptual content, interpretations, and conclusions were developed by the authors.
